# Lipoprotein(a) hyperlipidemia as cardiovascular risk factor: pathophysiological aspects

**DOI:** 10.1007/s11789-015-0074-0

**Published:** 2015-02-24

**Authors:** Gerd Schmitz, Evelyn Orsó

**Affiliations:** Institute for Laboratory Medicine and Transfusion Medicine, University Hospital of Regensburg, Franz-Josef-Strauss-Allee 11, 93053 Regensburg, Germany

**Keywords:** Lipoprotein(a), Cardiovascular risk, Pathophysiology, Biomarker, Lipoprotein(a), Kardiovaskuläres Risiko, Pathophysiologie, Biomarker

## Abstract

Lipoprotein (a) [Lp(a)] is a modified LDL particle with an additional apolipoprotein [apo(a)] protein covalently attached by a thioester bond. Multiple isoforms of apo(a) exist that are genetically determined by differences in the number of Kringle-IV type-2 repeats encoded by the LPA gene. Elevated plasma Lp(a) is an independent risk factor for cardiovascular disease.

The phenotypic diversity of familial Lp(a) hyperlipidemia [Lp(a)-HLP] and familial hypercholesterolemia [FH], as defined risks with genetic background, and their frequent co-incidence with additional cardiovascular risk factors require a critical revision of the current diagnostic and therapeutic recommendations established for isolated familial Lp(a)-HLP or FH in combination with elevated Lp(a) levels.

Lp(a) assays still suffer from poor standardization, comparability and particle variation. Further evaluation of the current biomarkers and establishment of novel comorbidity biomarkers are necessary for extended risk assessment of cardiovascular disease in FH or Lp(a)-HLP and to better understand the pathophysiology and to improve patient stratification of the Lp(a) syndrome complex.

Lp(a) promotes vascular remodeling, increased lesion progression and intima media thickening through induction of M1-macrophages, antiangiogenic effects (e.g. vasa vasorum) with secretion of the antiangiogenic chemokine CXCL10 (IP10) and CXCR3 mediated activation of Th1- and NK-cells.

In addition inhibition of serine proteases causing disturbances of thrombosis/ hemostasis/ fibrinolysis, TGFb-activation and acute phase response (e.g. CRP, anti-PL antibodies) are major features of Lp(a) pathology. Anti-PL antibodies (EO6 epitope) also bind to oxidized Lp(a).

Lipoprotein apheresis is used to reduce circulating lipoproteins in patients with severe FH and/or Lp(a)-HLP, particularly with multiple cardiovascular risks who are intolerant or insufficiently responsive to lipid-lowering drugs.

## Abstract (de)

Lipoprotein (a) [Lp(a)] ist ein modifizierter LDL-Partikel assoziiert mit Apolipoprotein [Apo(a)], das an den LDL-Partikel durch eine kovalente Thioesterbindung gebunden ist. Es sind mehrere genetisch determinierte Apo(a)-Isoformen bekannt, die sich durch die Anzahl der Typ-2 Kringle-IV Domänen unterscheiden.

Eine erhöhte Lp(a) Plasmakonzentration ist ein unabhängiger Risikofaktor für kardiovaskuläre Erkrankungen.

Aufgrund der phänotypischen Heterogenität der familiären Lp(a)-Hyperlipoproteinämie [Lp(a)-HLP] und der familiären Hypercholesterinämie [FH], als genetisch-definierte Risiken, sowie deren häufige Koinzidenz mit weiteren kardiovaskulären Risikofaktoren, müssen die aktuellen diagnostischen und therapeutischen Empfehlungen nochmals überprüft werden, die für die isolierte Lp(a)-HLP oder FH ohne erhöhtes Lp(a), bzw. deren Kombination mit erhöhten Lp(a)-Spiegeln etabliert wurden.

Die derzeit am Markt befindlichen Lp(a)-Bestimmungsmethoden sind ungenügend standardisiert, nicht exakt vergleichbar und berücksichtigen nicht die Variabilität der Partikelgröße. Die aktuellen Biomarker erfordern eine Ergänzung und Validierung durch neue Komorbiditäts-Marker, die die Pathogenese der kardiovaskulären Risikofaktoren bei FH oder Lp(a)-HLP präziser beschreiben und eine bessere Stratifizierung von Patienten mit Lp(a)-Syndromen ermöglichen.

Lp(a) fördert die vaskuläre Remodellierung, die zu einer Progression der atherosklerotischen Läsion und Verdickung der vaskulären Intima durch Induktion von M1-Makrophagen führen, sowie damit assoziierte antiangiogenetische Prozesse (z. B. Vasa vasorum) wie die Sekretion des anti-angiogenischen Chemokins CXCL10 (IP10) und des Th1- und NK-Zellen aktivierenden Chemokins CXCR3.

Darüber hinaus hemmt Lp(a) verschiedene Serinproteasen, die Störungen im Thrombose/Hämostase/Fibrinolyse-System verursachen können. Weitere pathogene Eigenschaften der Lp(a) sind die TGFb-Aktivierung und die Induktion einer Akute-Phase-Response Reaktion (z. B. CRP-Erhöhung, Synthese von Anti-PL-Antikörpern). Anti-PL-Antikörper (EO6 Epitope) binden bevorzugt an oxidierte Lp(a).

Die therapeutische Lipoprotein-Apherese wird bei schwerwiegenden FH- und/oder Lp(a)-HPL-Fällen zur Reduktion der zirkulierenden Lipoproteinkonzentration eingesetzt. Insbesondere Patienten mit multiplen Risikofaktoren, mit Medikamentenunverträglichkeit von Lipidsenkern oder mit unzureichender Lipidsenkung sollten mit der Lipidapherese behandelt werden.

## Introduction

Atherosclerotic cardiovascular disease (CVD) is one of the major factors for morbidity and mortality in Western countries. Established risk factors for CVD, include elevated plasma concentration of lipoprotein(a) (Lp(a)), high plasma concentration of low density lipoprotein (LDL), low plasma concentration of high density lipoprotein (HDL), hypertension, diabetes, obesity, metabolic syndrome and smoking among others [1–3 and references therein]. Since the atherogenic process leading to CVD begins in childhood and is progressive throughout the life span [2], screening for risk factors, appropriate patient stratification and therapeutic management are essential. Lp(a)-hyperlipidemia (Lp(a)-HLP) has been reported as an independent risk factor for CVD [4–6 and references therein]. Lp(a)-HLP occurs either alone, or in combination with other genetic backgrounds for hyperlipidemia and CVD risk, such as familial hypercholesterolemia (FH) or apolipoprotein E4-allele, therefore early recognition of diverse co-morbidities is essentially required [3]. However, despite of considerable efforts, the precise pathophysiological mechanisms, how Lp(a) contributes to atherogenesis, are not fully elucidated.

## Lipoprotein(a) hyperlipidemia (Lp(a)-HLP)

Lipoprotein(a) [Lp(a)], first described by Berg in 1963 [7], is composed of apolipoprotein (a) (apo(a)), covalently attached to apolipoprotein B-100 (apoB-100) by a disulfide bond on kringle-IV type-9 (KIV_9_) in close proximity to the LDL receptor binding site of apoB-100 [8]. The apo(a) gene (LPA) is a major determinant of the plasma concentration of Lp(a), and several genetic LPA-variants have been described, including variations in the kringle region-coding repeats, which account for the size polymorphism of apo(a), diverse single nucleotide polymorphisms (SNPs) and other variants in the promoter of LPA [9–11]. There is an inverse relationship between the number of kringle repeats of apo(a) and the concentration of Lp(a) in plasma (reviewed in 5). The plasma levels of Lp(a) show significant diversity in ethnical groups (e.g. African-Americans have higher plasma Lp(a) concentrations than Caucasians), and in individuals carrying apo(a) even of the same size polymorphism [10, 11], but the underlying mechanisms have yet to be identified. The latter issue implies the possibility of the presence of additional factors for CVD risk of Lp(a).

## Measurement of Lp(a) in plasma

Quantitative determination of Lp(a) in human plasma (or serum) is currently a standard approach of clinical laboratories. In contrast to first generation immunoassays, recognizing kringle-IV type-2 (KIV_2_), current diagnostic antibodies bind to KIV_9_, and this binding site is more stable than KIV_2_, even in frozen samples [12]. Taking into account that the molecular weight of apo(a), depending on the genetic polymorphisms of apo(a), varies between 275 and 800 kDa in different individuals [5], plasma levels of Lp(a) expressed in mg/L or mmol/L may lead to confusion. At present, there is no commercial Lp(a) assay that is completely insensitive to the variability in Lp(a) particle mass, and Lp(a) mass refers to the entire mass of the whole particle, including lipids, proteins and carbohydrates [13]. In their recent paper McConnell and co-workers claim for novel mass-insensitive Lp(a) assays, since lipoprotein “particle number” seems to be superior to component-based metrics for CVD risk prediction [13]. In a very recent work the simultaneous quantitation and size characterization of apo(a) by ultra-performance liquid chromatography/mass spectrometry was reported [14]. The latter may be an initial step for development of mass-insensitive Lp(a) assays in the future.

## Lipoprotein(a) in atherogenesis

The kringle domains of apo(a) show high degree of homology to the kringle-IV and kringle-V of plasminogen, the zymogen for the fibrinolytic serine protease plasmin, and the atherogenic potential of Lp(a) is partially attributed to this structural homology [reviewed in 15]. The kringle structure of Lp(a) and plasminogen differs in one single amino acid (R560S), and it protects apo(a) from enzymatic cleavage by plasminogen activators, such as tissue-type plasminogen activator (t-PA) and urokinase plasminogen activators (u-PA) [9, 15]. In addition to this molecular mimicry of Lp(a) to plasminogen, there are further mechanisms contributing to the role of Lp(a) in atherogenesis and development of CVD: (1) Lp(a) is able to bind to the tissue factor pathway inhibitor (TFPI), which is responsible for the prothrombotic properties of Lp(a) [16]. (2) Lp(a) can dock on certain lipoprotein receptors, including LDL receptor (LDLR), LDLR-related protein(s) and very low density lipoprotein (VLDL) receptor, leading to Lp(a) internalization, although to a lesser extent than the natural ligands of these receptors [17]. (3) Lp(a) can be entrapped by matricellular proteins (e.g. laminin, thrombospondin, tetranectin, fibrinogen/fibrin, glycosaminoglycans, fibronectin), leading to retention of Lp(a) and recruitment of monocytes in atherosclerotic lesions [15, 18]. (4) Lp(a) leads to endothelial barrier dysfunction through dysregulated myosin light chains via a Rho/Rho-kinase mediated signalling, and in this process a strong lysine-binding site in kringle-IV type-10 (KIV_10_) of apo(a) plays an essential role [19]. (5) Lp(a) modulates inflammatory responses and abolishes recruitment of neutrophils [20]. (6) Lp(a) promotes the differentiation of pro-inflammatory, M1-type macrophages, that secrete CXCL10 (IP10) chemokine leading to activation of T-helper-1 (Th1) lymphocytes and natural killer (NK) cells [21–23]. The chemokine CXCL10 is increasingly regarded as a potent inhibitor of angiogenesis, that may affect vasa vasorum angiogenesis. Thus Lp(a) may perpetuate vascular remodelling by blocking myoepithelial organization of newly formed vascular tubes. The importance of CXCL10 in vascular disease progression is further supported by the expanding literature.

In recent years Witztum and co-workers have demonstrated in consecutive papers that a substantial portion of pro-inflammatory and pro-atherogenic oxidized phospholipids, produced in response to reactive oxygen species (ROS), is present on apo(a) and Lp(a) [24 and references therein]. By using epitope-specific monoclonal antibodies (particularly E06, but also others), recognizing oxidized but not native phospholipids (i.e. oxidation-specific epitopes, OSEs), they have found plasma levels of oxidized phospholipids correlating with concentrations of Lp(a) in plasma, apo(a) size polymorphism and CVD [24]. The E06 antibody was able to inhibit the recognition and uptake of oxidized phospholipids and apoptotic cells by macrophage scavenger receptors, and reduce the progression of CVD in an atherosclerosis-susceptible mouse model [reviewed in 24]. The innate immune system recognizes OSEs as danger-associated molecular patterns (DAMPs), and uses innate pattern recognition receptors and soluble factors for their clearance [25 and references therein]. The E06 immunoreactivity of apo(a), indicating pro-inflammatory oxidized phospholipids accessible to DAMPs, was found to be strongly influenced by KIV_10_, which may explain the atherogenic potential of Lp(a) [26]. In addition, Lp(a) and oxidized LDL (oxLDL) both bind monocyte chemoattractant protein-1 (MCP-1/CCL2), a major chemokine in induction and progression of vascular inflammation, and oxidized phospholipids were shown to be major determinants for MCP-1 binding [27]. Based on the critical role of oxidized phospholipids and OSEs in Lp(a)-associated atherogenesis, development of atheroprotective vaccines recognizing and masking OSEs may be beneficial to reduce CVD risk.

The levels of oxidized phospholipids are modified by the activity of lipoprotein-associated phospholipase A2 (Lp-PLA_2_, former platelet activating factor acetylhydrolase, PAFAH), which converts oxidized phospholipids to oxidized fatty acids and lysophosphatidylcholine (lysoPC) [24, 28]. Both lipid species have been shown to be pro-inflammatory and pro-atherogenic *in vitro*. Stronger association of Lp-PLA_2_ was found with Lp(a) as compared to LDL [28].

In a recent study release of phosphatidylcholine-containing oxidized phospholipids and oxidized cholesteryl esters, such as oxidized derivatives from cholesteryl linoleate and cholesteryl arachidonate, was demonstrated downstream from obstructive plaques in human, and these bioactive lipids were captured during percutaneous interventions [29]. This study clearly shows the presence of critical pro-inflammatory and pro-atherogenic lipids in the local microcirculation in human arterial tissue.

Taken together, Lp(a) exerts its pro-atherogenic role via multiple mechanisms, and the precise compositional analysis of Lp(a) particles contributes to a sophisticated evaluation of Lp(a)-associated CVD risk.

## Lp(a)-lowering therapy

Currently it is unclear if lowering Lp(a) levels will also reduce the risk of CVD, since the first specific Lp(a) decreasing compound(s) have recently been developed and clinical outcome data have not yet been published [6].

Several previous pharmacological approaches have shown Lp(a)-lowering effects (e.g. nicotinic acid, oestrogen, eprotirome, antisense apoB-100, inhibitors of cholesteryl ester transfer protein (CETP), monoclonal antibodies against apo(a) or proprotein convertase subtilisin/kexin type-9 (PCSK9)), but the decrease of plasma Lp(a)-levels in Lp(a)-HLP are/were insufficient without major adverse effects [reviewed in 6].

Prevention and/or adequate treatment of additional CVD risks such as smoking, obesity, hypertension, diabetes, alcohol consumption, although they are important interventions, have cno major impact in the management of Lp(a)-HLP [1, 2].

The long-time use of Lp(a) apheresis in combination with maximal tolerated doses of lipid-lowering drugs reduced the plasma concentrations of Lp(a) by over 70 %, parallel with a decrease of the mean annual rate of major adverse coronary events, as a measure of outcome, in a recent longitudinal, multicenter, cohort study [30].

The recommendations of the HEART-UK Working Group indicates that patients with progressive CVD, and with plasma concentrations of Lp(a) above 60 mg/dL, and plasma LDL cholesterol above 3.2 mmol/L (despite of maximal lipid-lowering drug therapy) should be considered for LDL apheresis [31]. Although LDL apheresis is able to provide reduced plasma concentrations of Lp(a), based on the structural similarities of LDL and Lp(a) particles, patients with isolated Lp(a)-HLP should rather be treated by Lp(a) apheresis [reviewed in 32].

The lack of clear guidance for the indication of Lp(a) apheresis treatment requires further efforts, but most apheresis centers work currently with the criteria of (i) 60 mg/dL Lp(a) in plasma as cut-off, and (ii) history of advanced and/or progressive CVD, and alternatively (iii) presence of additional risk factors for CVD that accelerate the course of disease [3].

Further studies are required, however, (a) to optimize management in order to reduce CVD risk associated with Lp(a)-HLP, and (b) to evaluate what other intermediate and/or high risk groups may benefit from Lp(a) screening [33].

## Conclusion

Despite of emerging data from clinical and experimental studies our current knowledge about the impact of Lp(a)-HLP in development of atherogenic CVD is still insufficient. A critical reappraisal of the current diagnostic and screening criteria as well as therapeutic recommendations is required. Further evaluation of recent, and establishment of novel biomarkers are necessary for appropriate risk assessment of CVD in Lp(a)-HLP. Novel mass-insensitive Lp(a) assays should be developed for precise laboratory screening of elevated plasma Lp(a), and follow-up of therapeutic effects in Lp(a)-HLP.
